# Sirtuin 5 is Dispensable for CD8^+^ T Cell Effector and Memory Differentiation

**DOI:** 10.3389/fcell.2021.761193

**Published:** 2021-12-13

**Authors:** Qianqian Duan, Jiying Ding, Fangfang Li, Xiaowei Liu, Yunan Zhao, Hongxiu Yu, Yong Liu, Lianjun Zhang

**Affiliations:** ^1^ Institute of Systems Medicine, Chinese Academy of Medical Sciences and Peking Union Medical College, Beijing, China; ^2^ Suzhou Institute of Systems Medicine, Suzhou, China; ^3^ School of Life Science and Technology, China Pharmaceutical University, Nanjing, China; ^4^ Institute of Biomedical Electromagnetic Engineering, Shenyang University of Technology, Shenyang, China; ^5^ Department of Systems Biology for Medicine, School of Basic Medical Sciences, Fudan University, Shanghai, China; ^6^ Cancer Institute, Xuzhou Medical University, Xuzhou, China; ^7^ Center of Clinical Oncology, The Affiliated Hospital of Xuzhou Medical University, Xuzhou, China; ^8^ Jiangsu Center for the Collaboration and Innovation of Cancer Biotherapy, Cancer Institute, Xuzhou Medical University, Xuzhou, China

**Keywords:** sirtuin 5 (SIRT5), CD8 T cell, memory T cell, infecion, effector T cell

## Abstract

CD8^+^ T cell effector and memory differentiation is tightly controlled at multiple levels including transcriptional, metabolic, and epigenetic regulation. Sirtuin 5 (SIRT5) is a protein deacetylase mainly located at mitochondria, but it remains unclear whether SIRT5 plays key roles in regulating CD8^+^ T cell effector or memory formation. Herein, with adoptive transfer of Sirt5^+/+^ or Sirt5^−/−^ OT-1 cells and acute *Listeria monocytogenes* infection model, we demonstrate that SIRT5 deficiency does not affect CD8^+^ T cell effector function and that SIRT5 is not required for CD8^+^ T cell memory formation. Moreover, the recall response of SIRT5 deficient memory CD8^+^ T cells is comparable with Sirt5^+/+^ memory CD8^+^ T cells. Together, these observations suggest that SIRT5 is dispensable for the effector function and memory differentiation of CD8^+^ T cells.

## Introduction

CD8^+^ T cells are the main immune effector cells, protecting host against viral/bacterial infection and tumor development. Upon antigen stimulation, naïve CD8^+^ T cells undergo extensive proliferation and acquisition of effector functions, which is followed by memory T cell formation. In response to acute infection, the memory formation of CD8^+^ T cells consists of the expansion phase, contraction phase, and memory formation and maintenance phase (Williams and Bevan, 2007). The activation of naïve CD8^+^ T cells is marked with the upregulation of multiple surface markers including CD25, CD44, CD69, and CD98 and downregulation of CD62L. Meanwhile, T cell activation process is accompanied by striking metabolic switch from oxidative phosphorylation to aerobic glycolysis, and glycolytic metabolism is the key required for acquisition of CD8^+^ T cell effector functions (Chang et al., 2013). Central memory CD8^+^ T cells (Tcm) display high level expression of CD62L, allowing their homing to lymph node (LN). Importantly, Tcm cells exhibit substantial mitochondrial spare respiratory capacity (SRC) and fatty acid oxidation (FAO), which allows to sustain their long-term survival and metabolically prepared for secondary expansion (Zhang and Romero, 2018). Indeed, accumulating evidence suggests that T cell activation and differentiation are coupled to metabolic reprogramming, and alterations of metabolic activity can determine the CD8^+^ T cell fate (Zhang and Romero, 2018).

The sirtuins possess NAD^+^-dependent protein deacetylase activity and contain seven members in mammalian cells with different subcellular localization and functions, the SIRT1∼SIRT7 (Houtkooper et al., 2012; Song et al., 2018). SIRT1, SIRT6, and SIRT7 are mainly located in the nucleus and SIRT3-5 in the mitochondria, whereas SIRT2 is predominantly located in the cytoplasm. Sirtuins have been shown to play important roles in regulating diverse key biological processes, including gluconeogenesis, glycolysis, the tricarboxylic acid (TCA) cycle, and lipid metabolism (Houtkooper et al., 2012). Yet, emerging studies demonstrated that sirtuin members were involved in diverse stages of immune response, and more detailed roles of sirtuins are currently under investigation. For instance, SIRT2 inhibits T cell metabolism by targeting multiple key enzymes, such as hexokinase, ATP-dependent 6-phosphofructokinase, aldolase, glyceraldehyde-3-phosphate dehydrogenase (GAPDH), enolase, 2-oxoglutarate dehydrogenase, and succinate dehydrogenase, through its deacetylase activity (Hamaidi et al., 2020). Of note, SIRT2 deficiency in T cells increases both glycolysis and oxidative phosphorylation to enhance their proliferation and anti-tumor effector functions. Moreover, SIRT1 regulates glycolytic activity in innate immune cells by cooperating with hypoxia-inducible factor–1α (HIF1α) to impact their functional differentiation (Yu et al., 2018). In addition, SIRT1 programs the differentiation of CD4^+^ T cells by driving the production of cytokine interleukin-12 (IL-12) and transforming growth factor–β1 in dendritic cell through a HIF1α–dependent signaling pathway (Liu et al., 2015). Therefore, sirtuins are likely novel therapeutic targets against tumor or other diseases *via* modulating metabolism or epigenetic activity.

Sirtuin 5 (SIRT5) is a unique member amongst the seven sirtuins, because it not only has deacetylase activity but also possesses stronger demalonylase, desuccinylase, and deglutarylase functions (Du et al., 2011; Tan et al., 2014; Wang et al., 2017; Kumar and Lombard, 2018). So far, thousands of potential substrates of SIRT5 have been identified *via* proteomic analysis including various critical metabolic enzymes involved in ketogenesis (Rardin et al., 2013), amino acid degradation, TCA cycle, fatty acid metabolism (Park et al., 2013), and glycolysis (Nishida et al., 2015). SIRT5 has been well recognized as a regulator of various metabolic processes, which was involved in multiple diseases such as DSS-induced colitis and hypertrophic cardiomyopathy (Rardin et al., 2013; Nishida et al., 2015; Sadhukhan et al., 2016; Kumar and Lombard, 2018). Previous studies indicated that the modulation of cellular metabolism plays a key role in dictating immune cell development and function (Norata et al., 2015). For instance, Wang et al. (2017) have revealed that SIRT5 reprograms the metabolism process of macrophage to repress the pro-inflammatory response by activating the PKM2 kinase activity and block macrophage IL-1β production. In addition, Jeng et al. (2018) reported that the metabolic reprogramming of resting memory CD8^+^ T cell is closely correlated with the loss of SIRT1. Given that SIRT5 is an important metabolic or epigenetic regulator, a better understanding of its roles in regulating CD8^+^ T cell immune response is needed.

Herein, our present study aimed to explore the effects of SIRT5 on the effector function and memory differentiation of CD8^+^ T cells with OT-1 TCR transgenic mice and acute *Listeria monocytogenes* infection model. To our surprise, we did not observe significant phenotypic changes regarding the activation, differentiation, and effector function of CD8^+^ T cells in the absence of SIRT5. Furthermore, SIRT5 deficiency did not affect the recall of memory CD8^+^ T cells. Together, although SIRT5 affected the mitochondrial function to some extent, it is dispensable for differentiation and function of CD8^+^ T cells.

## Methods

### Animal

Female C57BL/6N mice (6–8 weeks old, WT) were purchased from Vital River Co., Ltd. (Beijing, China). B6;129-Sirt5^tm1Fwa^/J (Sirt5^−/−^) mice were kindly provided by Prof. Hongxiu Yu and were described by Wang et al. (2017). CD45.1^+^ OT-1 TCR transgenic mice on a C57BL/6 background were housed under specific pathogen-free conditions in the animal facility of Suzhou Institute of Systems Medicine (Suzhou, China). CD45.2^+^ Sirt5^−/−^ mouse was crossed with CD45.1^+^ OT-1 mouse to obtain CD45.1/2^+^ Sirt5^+/−^ OT-1 and CD45.1/2^+^ Sirt5^+/−^ offspring mice, and then, the CD45.1/2^+^ Sirt5^+/−^ OT-1 mouse was crossed with CD45.1/2^+^ Sirt5^+/−^ mouse to produce CD45.1^+^ Sirt5^+/+^ OT-1, CD45.1/2^+^ Sirt5^+/+^ OT-1, CD45.1^+^ Sirt5^−/−^ OT-1, CD45.1/2^+^ Sirt5^−/−^ OT-1, and other genotype offspring mice. *Listeria* infection experiments were performed in Animal Biosafety Level-2 laboratories.

### CD8^+^ T Cell Activation and *in vitro* Culture

CD8^+^ T cells were sorted from the spleens of Sirt5^+/+^ OT-1 mice and Sirt5^−/−^ OT-1 mice by the Mouse CD8^+^ Naïve T Cell Isolation Kit (BioLegend, no. 480044). CD8^+^ T cells (2 × 10^6^) were plated into each well of 24-well plate in 2-ml medium, which was precoated with αCD3 (Invitrogen, no. 16-0031-86) and αCD28 (Invitrogen, no. 16-0281-86) antibody. IL-2 (PeproTech, no. 200-02) was added into the culture medium, and the final concentration was 10 ng/ml. The activation phenotype of CD8^+^ T cells was assessed at the time points of 6 h, 24 h, 72 h, and 6 days after activation. On third day, dead cells were removed by Ficoll-Paque (GE Healthcare, no. 17-1440-03), and the rest live CD8^+^ T cells were continually cultured in the medium containing IL-2 (10 ng/ml) and IL-7 (10 ng/ml) (PeproTech, no. 200-07). On the sixth day, CD8^+^ T cells were collected to measure the cytokine secretion ability by restimulation with N4 peptide.

### Quantitative Polymerase Chain Reaction

Quantitative PCR analysis was performed according to a previously described method (Duan et al., 2019). β-actin was used as the internal reference. Four technical replicates were performed. The following primers were used: SIRT5 forward primer: 5- GTC​ATC​ACC​CAG​AAC​ATC​GA-3, SIRT5 reversed primer: 5- ACG​TGA​GGT​CGC​AGC​AAG​CC-3 (Ogura et al., 2010), and β-actin forward primer: 5-GGG​CTA​TGC​TCT​CCC​TCA​C-3, β-actin reversed primer: 5-GAT​GTC​ACG​CAC​GAT​TTC​C-3 (Cheeran et al., 2007).

### Adoptive Naïve T Cell Transfer and Bacterial Infection

Sirt5^+/+^ and Sirt5^−/−^ naïve OT-1 cells were sorted using the Mouse CD8^+^ Naïve T Cell Isolation Kit (BioLegend, no. 480044). CD45.1 and CD45.2 are allelic variants of CD45 expressed in all leukocytes including CD8^+^ T cell and can be efficiently distinguished by flow cytometry. In the adoptive naïve T cell transfer experiments, CD45.1 and CD45.2 were used to be as congenic marker to distinguish donor CD8^+^ T cells and host CD8^+^ T cells. The recipients were all CD45.2^+^ mice, and the transferred naïve CD8^+^ T cells were CD45.1^+^ or CD45.1/2^+^ OT-1 cells. For separate transfer of Sirt5^+/+^ and Sirt5^−/−^ naïve OT-1 cells, 5 × 10^4^ cells were transferred into naïve host intravenously (i.v.), separately. For co-transfer of Sirt5^+/+^ and Sirt5^−/−^ naïve OT-1 cells, a total of 1 × 10^5^ cells was transferred i.v., as the amount of Sirt5^+/+^ and Sirt5^−/−^ naïve OT-1 cells were both 5 × 10^4^. Each mouse was injected i.v. 2,000 CFU *Listeria monocytogenes* stably expressing ovalbumin (LM-OVA) at primary infection. Co-transferred mice were used for recall experiments and were injected i.v. 1 × 10^4^ CFU LM-OVA on the 40th day at secondary infection.

### Flow Cytometry (Surface and Intracellular Staining)

Single-cell suspensions obtained from the blood, spleen, and lymphocyte node were used for flow cytometry analysis. For cell surface staining, cells were first performed by viability staining with Fixable Viability Dye eFluor 506 (eBioscience, no. 65-0866-18) for 20 min on ice. Then, the cells were surface stained for 25 min on ice: anti-CD8 (Brilliant Violet 711, BioLegend, no. 100748), anti-CD25 (PB, 102022, no. 100748), anti-CD44 (APC-Cy7, BioLegend, no. 103028), anti-CD69 (FITC, BioLegend, no. 104506), anti-CD98 (Alexa Fluor 647, BioLegend, no. 128210), anti-CD62L (PE or APC, BioLegend, no. 104408 or 104412), anti-CD45.1 (FITC or Percp/Cy5.5, BioLegend, no. 110706 or 110728), anti-CD45.2 (Pacific Blue, BioLegend, no. 109820), anti-KLRG1 (PE-Cy7, Invitrogen, no. 25-5893-82), and anti-CD127 (PE, BioLegend, no. 135010). For intracellular cytokine staining, cells were first fixed with Fixation Buffer (BioLegend, no.420801) for 20 min on ice and permeabilized with Permeabilization Buffer (BioLegend, no.421002). Then, cells were stained with anti–IL-2 (PE, BioLegend, no. 503808), anti–tumor necrosis factor–α (TNFα) (FITC, BioLegend, no. 506306), and anti–interferon-γ (IFNγ) (APC, BioLegend, no. 505810) for 25 min on ice. For intracellular transcription factor staining, cells were first fixed with eBioscience Foxp3/Transcription Factor Staining Buffer (Invitrogen, no.00-5223-56 and 00-5123-43), permeabilized with Permeabilization Buffer (Invitrogen, no.00-8333-56), and then were stained with anti-Tcf1 (Alexa Fluor 647, BioLegend, no. 655204) and anti-T-bet (PE-Cy7, BioLegend, no. 644824). The stained cell samples were resuspended in FACS buffer (phosphate-buffered saline containing 2% fetal bovine serum) and loaded in an LSR Fortessa flow cytometer (Becton Dickinson, San Jose, CA). The FCA data were analyzed by FlowJo software.

### Mitochondrial Potential Measurement by Flow Cytometry

Mitochondrial mass and activity were assessed by MitoTracker green (MTG) (Invitrogen, no. M7514) and tetramethylrhodamine ethyl ester (TMRE) (Invitrogen, no. T669) staining, respectively. In brief, cells were stained with MTG and TMRE at 37°C in dark for 30 min, which was followed by incubation with Fixable Viability Dye eFluor 506 for 20 min and subsequent cell surface staining. Finally, cells were resuspended in FACS buffer for further analysis.

### *In vitro* Restimulation

Single-cell suspensions obtained from the blood, spleen, and lymphocyte node were used for restimulation *in vitro* to measure cytokine secretion ability. At indicated time points, single-cell suspensions were seeded into 96-well plate. The cells were pre-stimulated with 1 μM SIINFEKL (N4) peptide (synthetized by China Abcepta Biotech Ltd., Co.) for 30 min. Then, cells were restimulated for another 4 h, and meanwhile, the medium was added eBioscience Brefeldin A (Invitrogen, no. 00-4506-51) and eBioscience Monensin Solution (Invitrogen, no. 00-4505-51).

### Western Blot Analysis

Western blot analysis was performed according to a previously described method (Duan et al., 2019). Total protein lysates were generated by lysing the purified CD8^+^ T cells from Sirt5^+/+^ and Sirt5^−/−^ OT-1 mice in RIPA buffer containing 50 mM Tris-HCl, 150 mM NaCl, 1% Triton X-100, 0.1% SDS, 1 mM EDTA, protease inhibitor cocktail tablet (Roche, no. 11697498001). The SIRT5 antibody was purchased from Cell Signaling Technology (no. 8782S), and β-actin was from ABclonal Technology (no. AC026).

### Statistical Analysis

Statistical analysis was performed with GraphPad Prism software. The comparison of two groups was done by two-tailed Student’s *t*-test. Data are presented as the mean ± SD. Difference was considered statistically significant when *p* < 0.05 (*p < 0.05; **p < 0.01; ***p < 0.001).

## Results

### SIRT5 Deficiency Does Not Impact the Activation and Functionality of CD8^+^ T Cells

**FIGURE 1:**
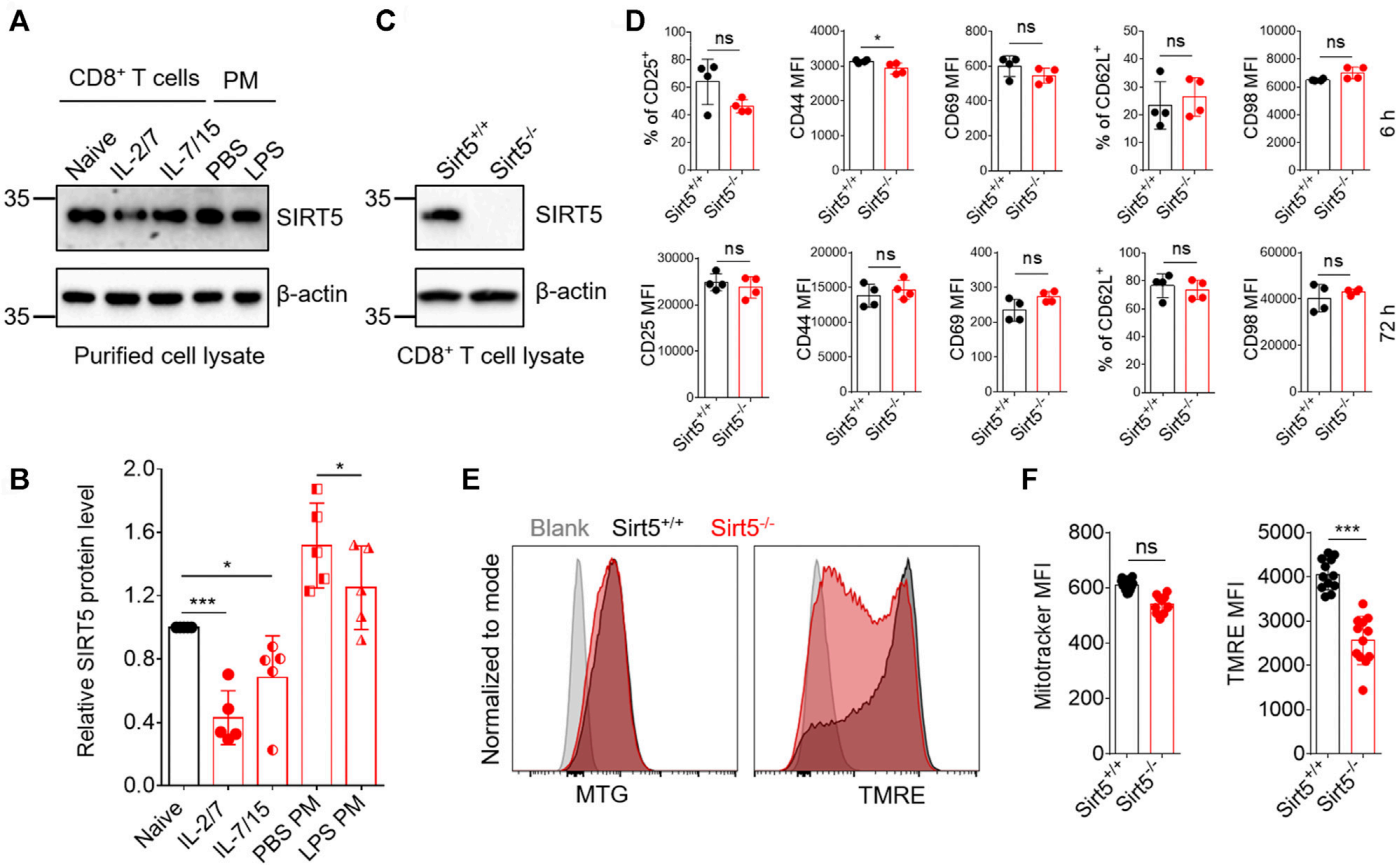
SIRT5 deficiency has no evident impacts on CD8^+^ T cell activation *in vitro*. **(A)** SIRT5 protein expression in purified effector or memory CD8^+^ T cells and peritoneal macrophages (PM). The activated CD8^+^ T cells were further induced by IL-2/7 or IL-7/15 for another 4 days, to induce the effector and memory fate, and the phenotypes were confirmed by flow cytometry analysis. PMs were obtained from mice which were injected ip with 2 ml of sodium thioglycollate 3 days before. The adherent macrophages were thus stimulated with LPS for 12 h. **(B)** Quantification of Western blot images for SIRT5 from five biological replicates. Mean ± SD (*n* = 5), Student’s *t*-test, *: *p* < 0.05; **: *p* < 0.01; ***: *p* < 0.001; ns, not significant. **(C)** Western blot analysis of SIRT5 expression in CD8^+^ T cells from Sirt5^+/+^ and Sirt5^−/−^ OT-1 mice. **(D)** Surface activation marker expression of Sirt5^+/+^ and Sirt5^−/−^ OT-1 cells at the time points of 6 and 72 h assessed by flow cytometry. **(E)** Representative histograms of MTG and TMRE of Sirt5^+/+^ and Sirt5^−/−^ OT-1 cells on the sixth day after activation. **(F)** Statistical analysis of mean fluorescence intensity (MFI) of MTG and TMRE. Mean ± SD (*n* = 12), Student’s *t*-test, ***: *p* < 0.001.

**FIGURE 2:**
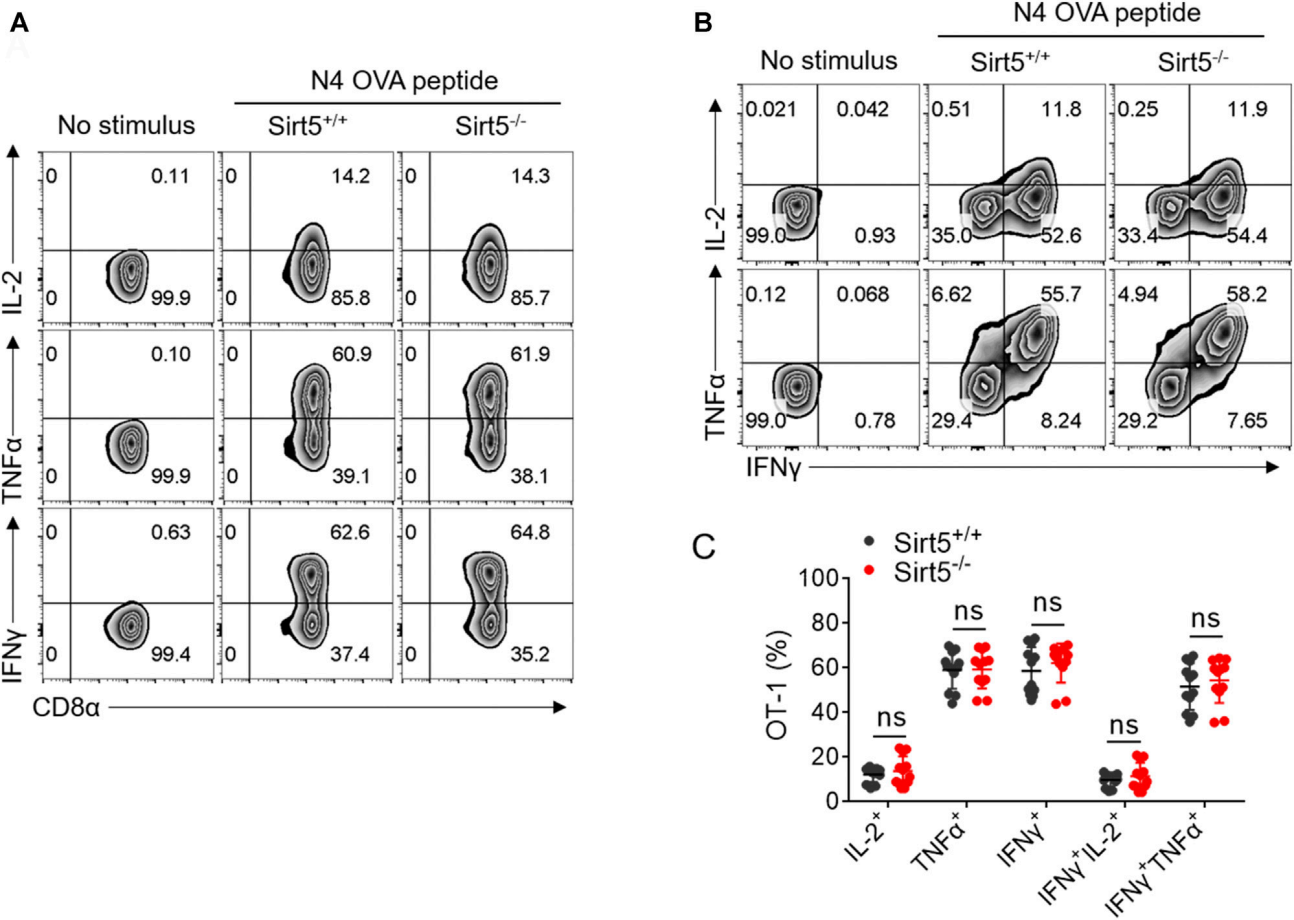
SIRT5 deficiency does not affect the cytokine secretion *in vitro* restimulation. **(A,B)** Representative dot plots of IL-2, TNFα, and IFNγ expression levels by flow cytometry on the sixth day after activation. **(C)** Statistical analysis of OT-1 cell ratio of cytokine experiment. Mean ± SD (*n* = 12), Student’s *t*-test.

First, we compared the expression pattern of SIRT5 at both mRNA and protein levels in different CD8^+^ T cell subtypes including naïve, effector, and memory CD8^+^ T cells and other cell types such as macrophages, where SIRT5 has been reported to play important roles (Wang et al., 2017) (Figures 1A,B; Supplementary Figure S1A). Results showed that SIRT5 protein levels were downregulated in the effector CD8^+^ T cells (cultured with IL-2/7) and CD8^+^ T cells with memory phenotype (cultured with IL-7/15) (Zoon, et al., 2017). In particular, we demonstrated that the mRNA expression of SIRT5 was also decreased in purified antigen-specific memory CD8^+^ T cells after bacterial infection (Supplementary Figure S1A), indicating the potential roles of SIRT5 in regulating CD8^+^ T cell effector and memory differentiation. Of note, the mRNA expression of SIRT5 was relatively lower in CD8^+^ T cells as compared to peritoneal macrophages. To characterize the precise role of SIRT5 in CD8^+^ T cells, Sirt5^−/−^ mice were crossed with OT-1 TCR transgenic mice on a C57BL/6 background to generate Sirt5^−/−^ OT-1 mice. With western blot analysis, we confirmed that SIRT5 was successfully knocked out in OT-1 mice (Figure 1C). The effects of SIRT5 on the activation phenotype of CD8^+^ T cells was assessed by measuring the surface marker expression at different time points. Flow cytometry analysis demonstrated that SIRT5 deficiency did not affect the expression pattern of CD25, CD44, CD69, CD98, and CD62L significantly (Figure 1D; Supplementary Figure S1B). In addition, we assessed the cytokine secretion ability of Sirt5^+/+^ and Sirt5^−/−^ OT-1 cells by *in vitro* restimulation but did not observe significant difference between Sirt5^+/+^ and Sirt5^−/−^ OT-1 cells (Figures 2A–C), which indicates that SIRT5 deficiency may not affect the CD8^+^ T effector function. As SIRT5 is mainly located in the mitochondria, it is unclear whether SIRT5 affects the mitochondrial mass and function of CD8^+^ T cells. Thus, we measured the mitochondrial mass with MTG staining and mitochondrial membrane potential with TMRE staining. SIRT5-deficient CD8^+^ T cells showed similar MTG MFI with that of Sirt5^+/+^ CD8^+^ T cells (Figures 1E,F), which is consistent with previously report that SIRT5 did not affect the mitochondrial mass (Buler et al., 2014). Mitochondrial membrane potential (_△_ψm) is a critical factor in the energy transformation and production and performs many non-energetic functions for mitochondria. Normal _△_ψm is a requisite for maintenance of mitochondrial function. Interestingly, SIRT5 deficiency in CD8^+^ T cells led to decreased TMRE MFI (Figures 1E,F), indicating of potential alteration of mitochondrial function in the absence of SIRT5.

### SIRT5 is Not Required for the Proliferation and Survival of CD8^+^ T Cells Upon Acute Infection

**FIGURE 3:**
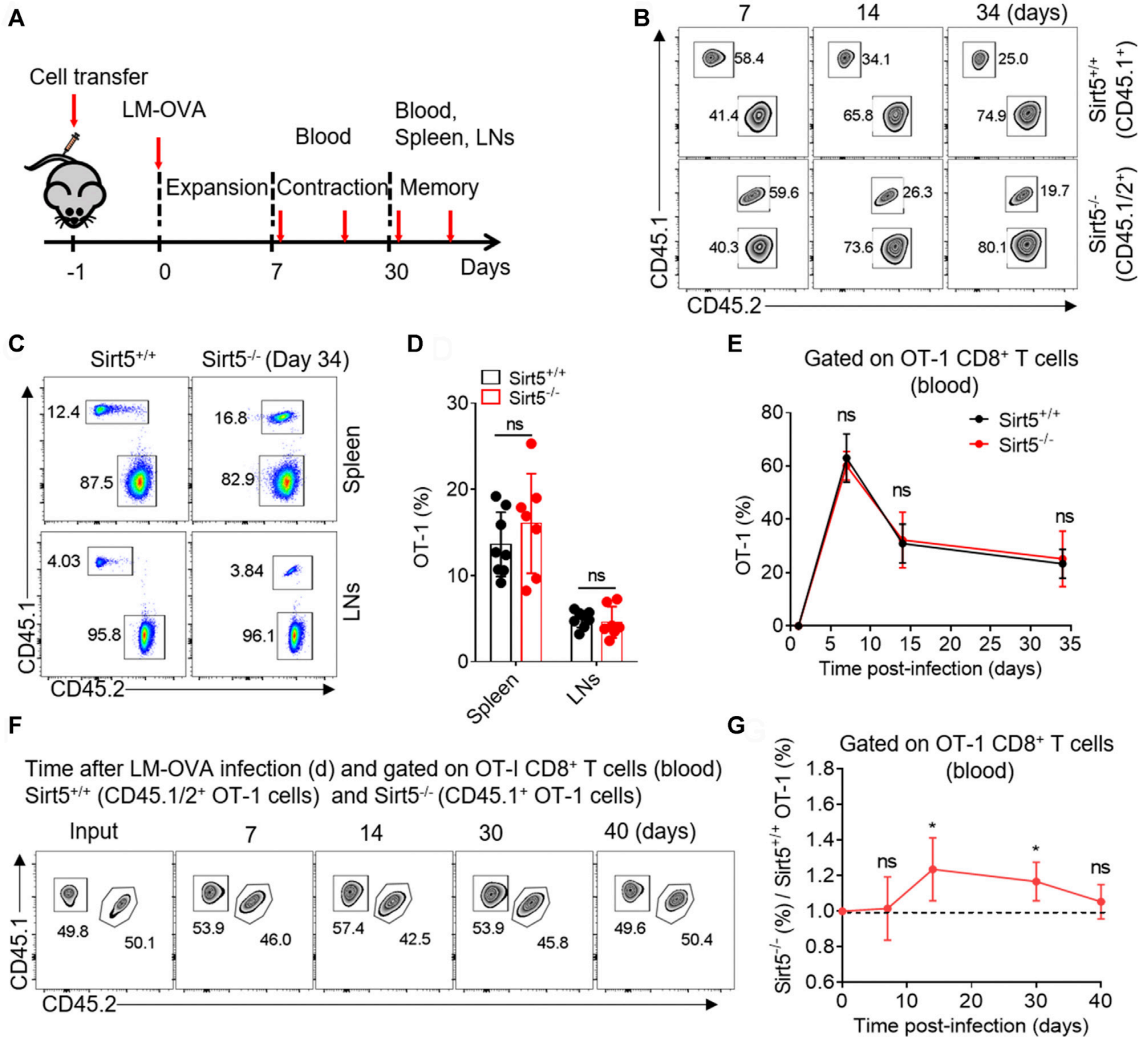
The effect of SIRT5 on the expansion and early memory formation of CD8^+^ T cells against acute infection. **(A)** Schematic representation of LM-OVA infection. Sirt5^+/+^ and Sirt5^−/−^ OT-1 cells are adoptively transferred into CD45.2^+^ naïve recipients followed by LM-OVA infection. Blood, spleen, and lymphocyte nodes (LNs) are obtained from the transferred mice at the indicated time points marked by red arrow. **(B)** Kinetics of the separately transferred OT-1 cells in the blood after primary infection. Mean ± SD (*n* = 9), Student’s *t*-test. **(C,D)** Percentage of the OT-1 cell population in the spleens and LNs on day 34 after infection. Mean ± SD (*n* ≥ 6), Student’s *t*-test. **(F,G)** Kinetics of the co-transferred OT-1 cells in the blood after primary infection. Mean ± SD (*n* = 6), Student’s *t*-test, *: *p* < 0.05.

SIRT5 is an important regulator of various energy metabolisms. Next, we want to know whether SIRT5 affects the expansion and the effector and memory differentiation of CD8^+^ T cells *in vivo*. We thus transferred Sirt5^+/+^ and Sirt5^−/−^ naïve OT-1 cells into wild-type mice i.v. and infected them with LM-OVA and then analyzed the proliferative response of OT-1 cells by flow cytometry at different time points during expansion, contraction, and memory maintenance phase of memory CD8^+^ T cell formation process (Figure 3A). We performed the kinetic analysis of Sirt5^+/+^ and Sirt5^−/−^ OT-1 cells in the blood of LM-OVA infected mice (Figures 3B,E). At the peak of expansion phase (seventh day after infection), we did not observe significant differences in the expansion of Sirt5^+/+^ and Sirt5^−/−^ OT-1 cells. The *in vivo* kinetic curves of Sirt5^+/+^ and Sirt5^−/−^ OT-1 cells were similar, suggesting that SIRT5 is unrelated to the proliferation and survival of CD8^+^ T cells. Next, we separated the spleens and lymphocyte nodes from infected mice and quantified their early memory CD8^+^ T cell of Sirt5^+/+^ and Sirt5^−/−^ OT-1 cells on the 34th day (Figures 3C,D). However, the ratio of early memory cell of Sirt5^−/−^ OT-1 cells in total CD8^+^ T subset was similar with that of Sirt5^+/+^ OT-1 cells. To avoid any effects of different host microenvironment, we carried out co-transfer mouse model experiment. Although Sirt5^−/−^ OT-1 cells had slight survival advantage during contraction phase (14 days after infection) in the co-transfer model, consistent with separate transfer model, they formed the comparable ratio of early memory cell with Sirt5^+/+^ OT-1 cells in memory maintenance phase (Figures 3F,G).

### SIRT5 Deficiency Does Not Impact Differentiation and Function of CD8^+^ T Cells *In vivo*

**FIGURE 4:**
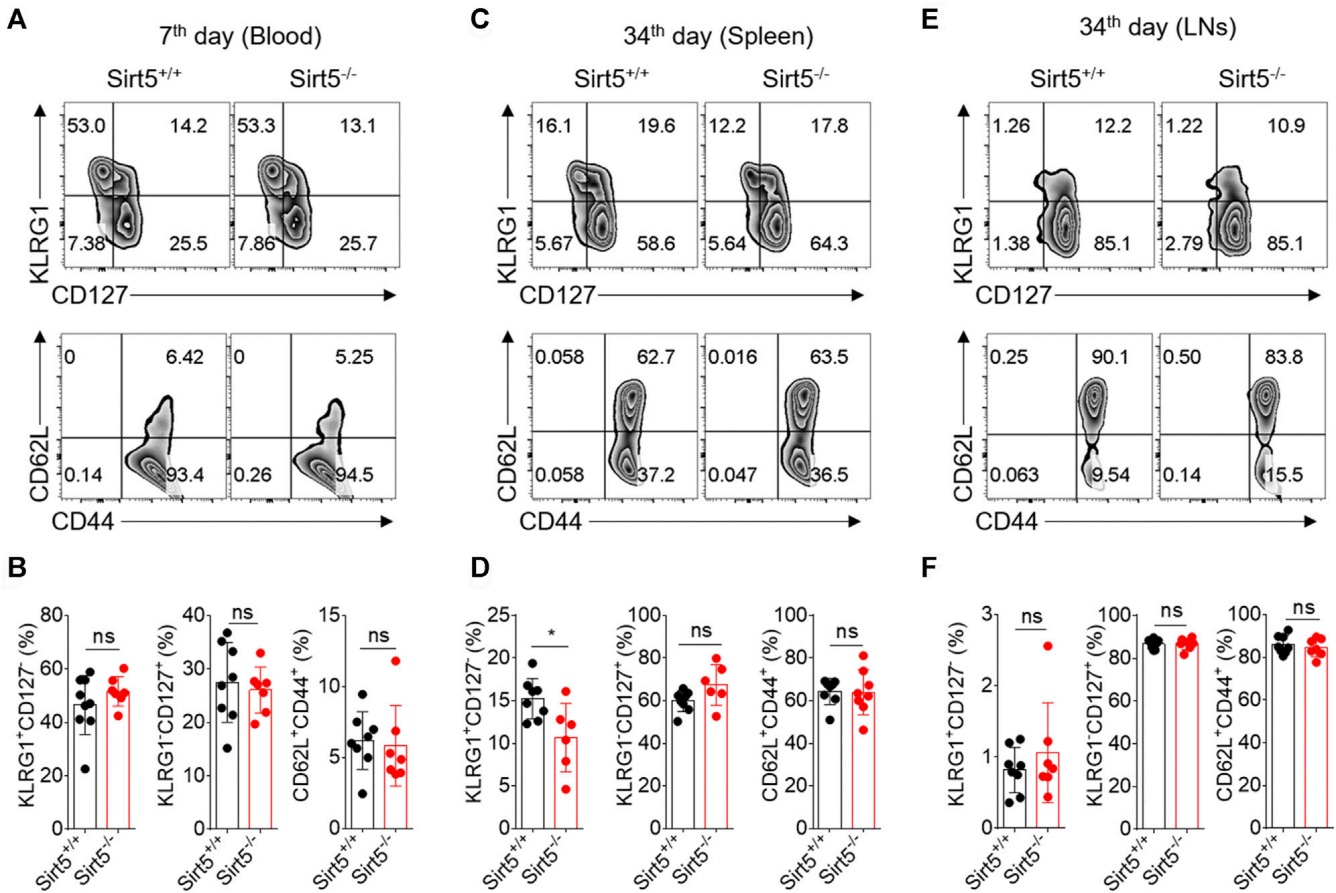
SIRT5 does not affect the differentiation of CD8^+^ T cells against the primary infection. **(A–C)** Percentage in the blood (day 7), spleen (day 34), and LNs (day 34) of the KLRG1^+^CD127^−^, KLRG1^-^CD127^+^, and CD62L^+^CD44^+^ populations at different time points after primary infection. **(D–F)** Corresponding population statistical diagram. Mean ± SD (*n* = 9), Student’s *t*-test, *: *p* < 0.05.

KLRG1, CD127, CD62L, and CD44 have been reported to define functionally distinct CD8^+^ T cell populations [short-lived effector cells (SLEC): KLRG1^+^CD127^−^, and memory-precursor effector cells (MPEC): KLRG1^−^CD127^+^; and central memory T cells (Tcm): CD62L^+^CD44^+^] (Bengsch et al., 2007; Sukumar et al., 2016; Renkema et al., 2020). Next, we measured the expression pattern of these surface markers by flow cytometry to characterize the OT-1 cell differentiation *in vivo*. The percentages of Tcm, SLEC, and MPEC population were all comparable between Sirt5^+/+^ OT-1 cells and Sirt5^−/−^ OT-1 cells on seventh day in blood of the separately transferred mice (Figures 4A,B). Consistently, there were no significant differences in these three subsets between the two genotypes in co-transfer model at different time points: 7th, 14th, 30th, and 40th day) (data not shown). Similarly, in the spleens and lymphocyte nodes, there was no statistical significance in Tcm and MPEC populations (Figures 4C–F). On the basis of these results, we concluded that SIRT5 deficiency may not affect the transition of OT-1 cells from effector to memory CD8^+^ T cells.

**FIGURE 5:**
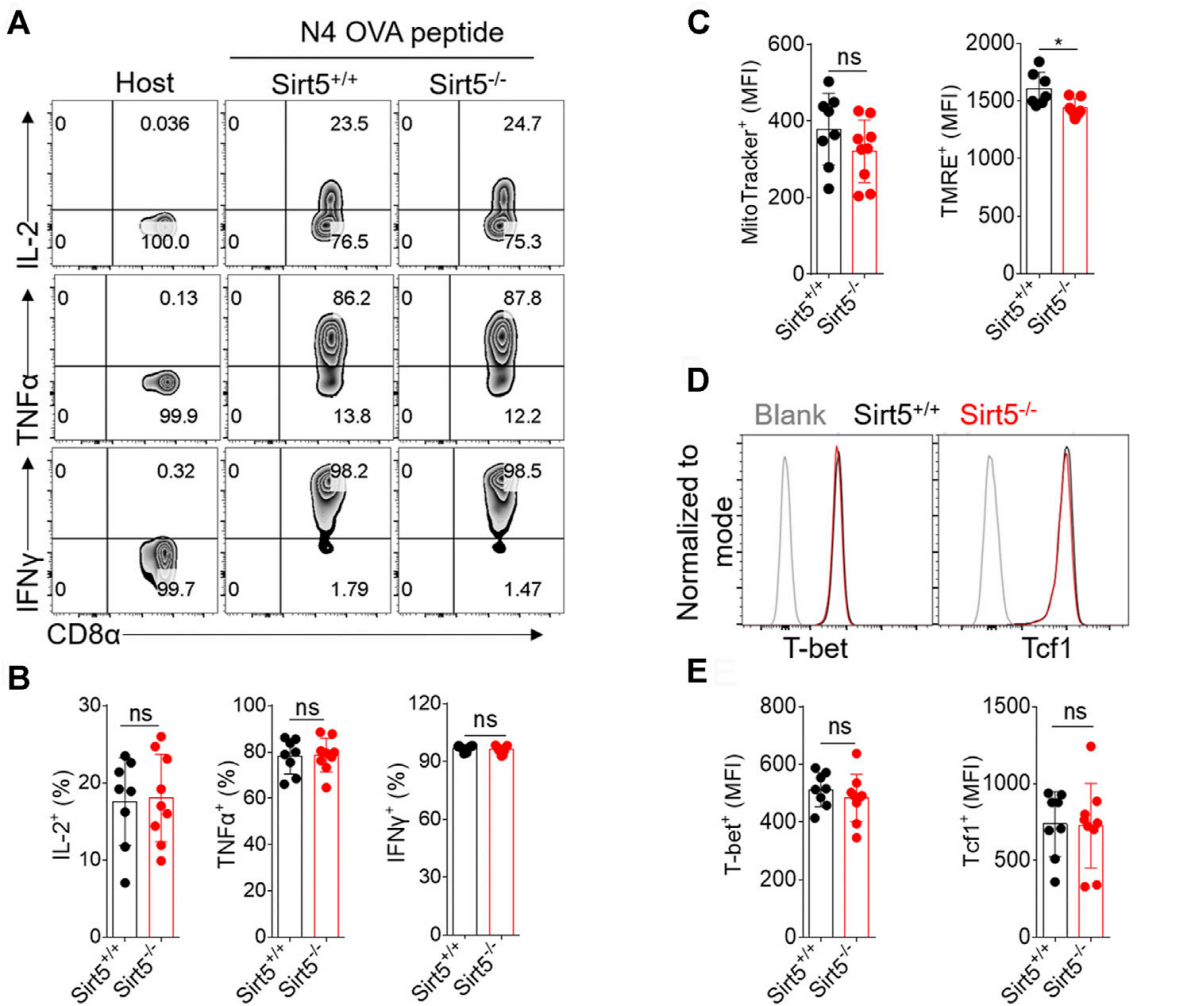
The effect of SIRT5 on the effector function, mitochondrial function, and transcription factor expression of CD8^+^ T cells from the spleens of transferred mice. **(A)** Representative dot plots of IL-2, TNFα, and IFNγ expression levels of OT-1 cells from the spleen tissues by flow cytometry *in vitro* restimulation. The transferred mice are sacrificed on day 34 after primary LM-OVA infection. **(B)** Statistical analysis of OT-1 cell ratio of cytokine experiment. Mean ± SD (*n* = 9), Student’s *t*-test. **(C)** Statistical analysis of MTG and TMRE MFI. Mean ± SD (*n* = 9), Student’s *t*-test, *: *p* < 0.05. **(D)** Representative histograms of Tcf1 and T-bet protein expression level of Sirt5^+/+^ and Sirt5^−/−^ OT-1 cells from the spleens. **(E)** Statistical analysis of Tcf1 and T-bet MFI. Mean ± SD (*n* = 9), Student’s *t*-test.

Next, we investigated whether SIRT5 affects the CD8^+^ T cell effector function *in vivo*. Yet, we observed comparable IFNγ, TNFα, and IL-2 production between Sirt5^+/+^ OT-1 cells and Sirt5^−/−^ OT-1 cells in the spleens and lymphocyte nodes (Figures 5A,B; Supplementary Figure S2). Consistently, the cytokine production capacity was comparable between the two genotypes in the blood of both separate transferred and co-transferred mice on the 7th, 14th, 30th, and 40th days (data no shown). In addition, we detected the mitochondria mass and function of OT-1 cells with MTG and TMRE staining in the spleens and lymphocyte nodes, and consistent with *in vitro* results, we found that SIRT5 did not affect the MFI of MTG, but decreased the TMRE MFI (Figure 5C). Tcf1 is a critical transcription factor required for CD8^+^ T cell memory formation and T-bet is highly expressed in the effector T cells; we thus detected the protein expression of transcription factors Tcf1 and T-bet (Figures 5D,E). However, SIRT5 did not significantly affect the protein expression of Tcf1 and T-bet.

### SIRT5 Deficient Memory CD8^+^ T Cells Mounted Comparable Recall Responses

**FIGURE 6:**
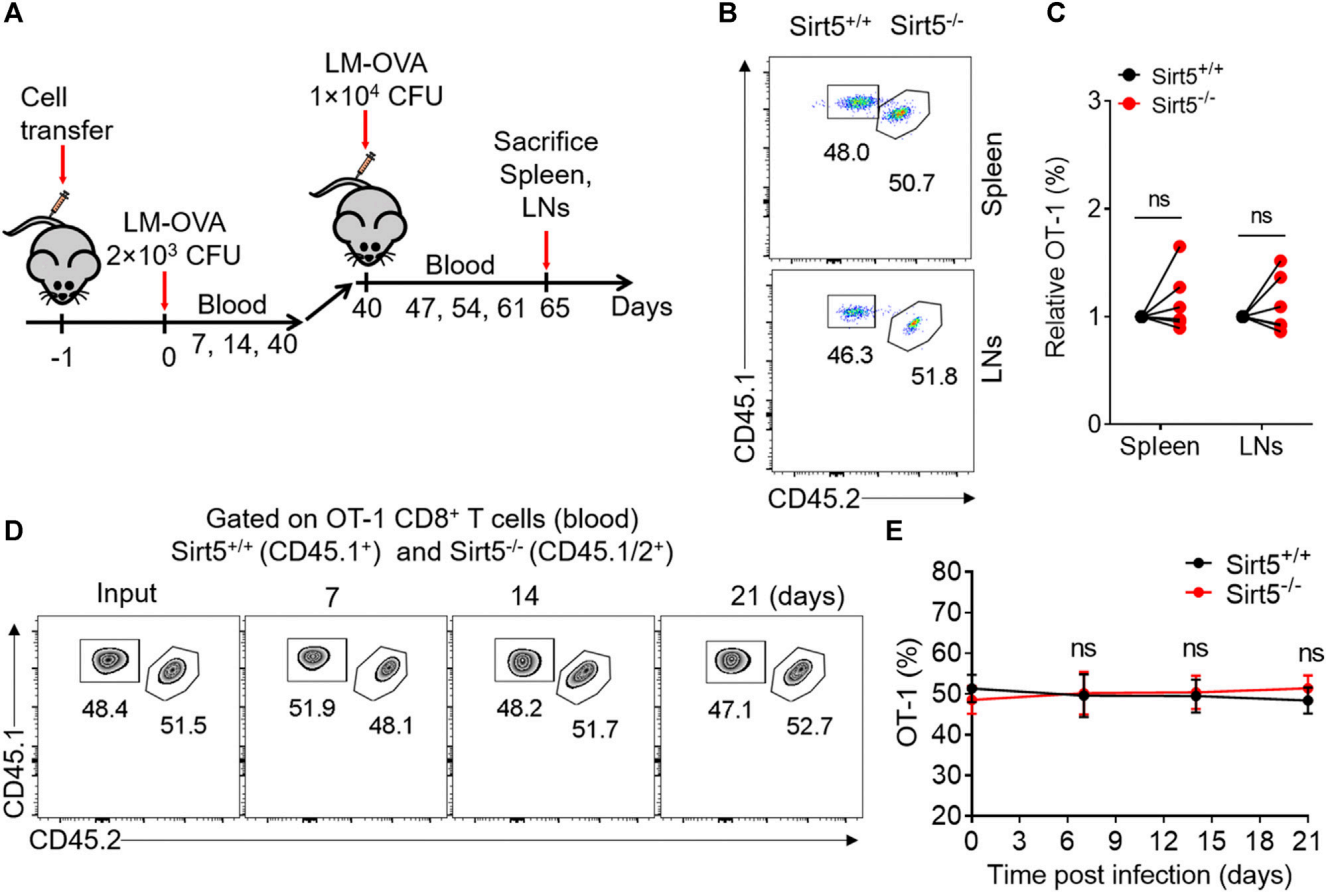
The role of SIRT5 in the expansion and survival of CD8^+^ T cells after secondary infection. **(A)** Schematic representation of LM-OVA infection. Sirt5^+/+^ and Sirt5^−/−^ OT-1 cells are co-transferred into CD45.2^+^ naïve recipients followed by LM-OVA infection. On day 40 after primary infection, these mice are infected with fivefold LM-OVA. **(B,C)** Percentage of the OT-1 cell population in the spleens and LNs on day 25 after secondary infection. Mean ± SD (*n* = 6), Student’s *t*-test. **(D,E)** Kinetics of the co-transferred OT-1 cells in the blood after secondary infection. Mean ± SD (*n* = 6), Student’s *t*-test.

**FIGURE 7:**
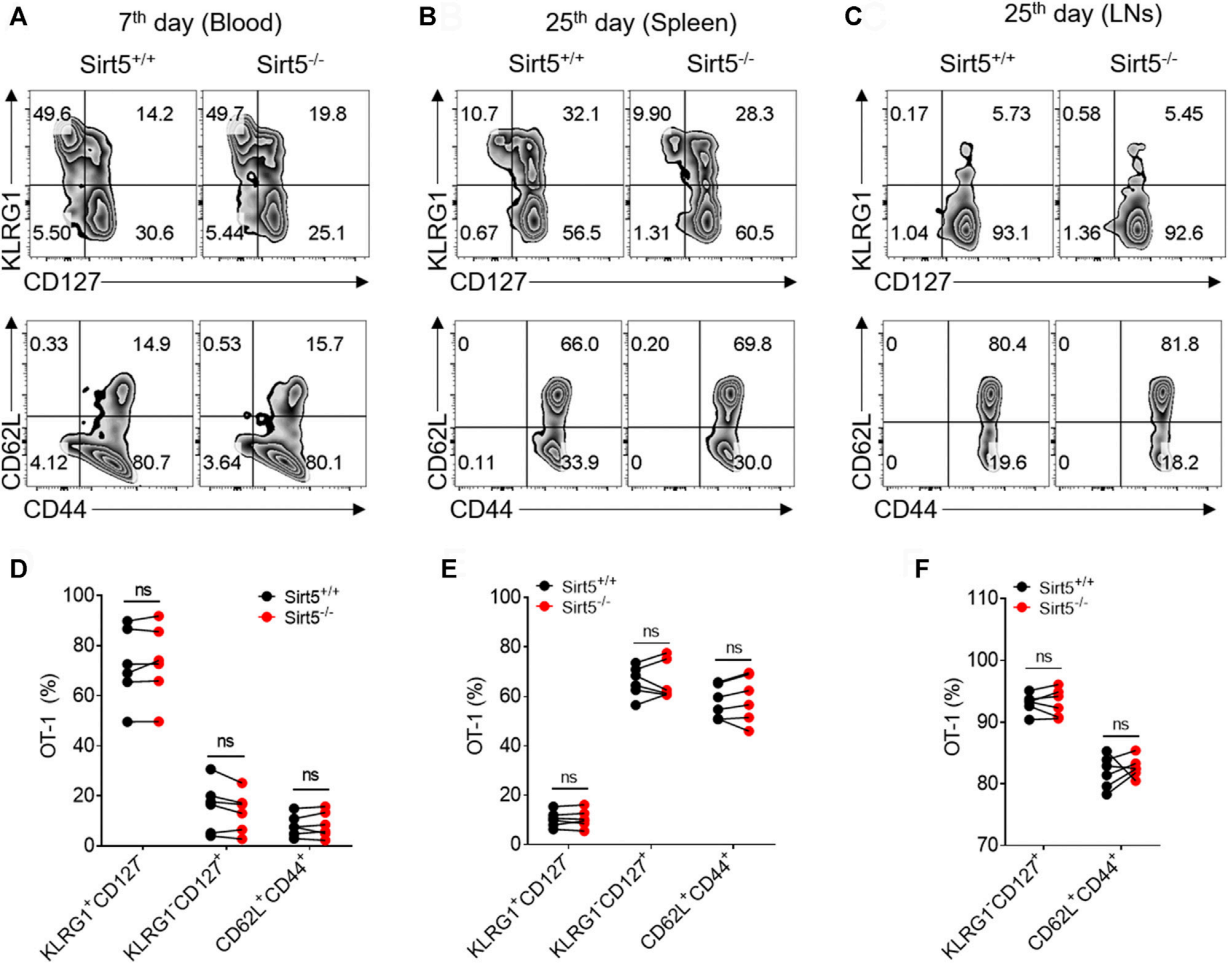
SIRT5 deficiency does not affect the differentiation of CD8^+^ T cells during the recall process. **(A–C)** Percentage in the blood (day 7), spleen (day 25), and LNs (day 25) of the KLRG1^+^CD127^−^, KLRG1^-^CD127^+^ and CD62L^+^CD44^+^ populations at different time points after secondary infection. **(D–F)** Corresponding population statistical diagram. Mean ± SD (*n* = 9), Student’s *t*-test.

**FIGURE 8:**
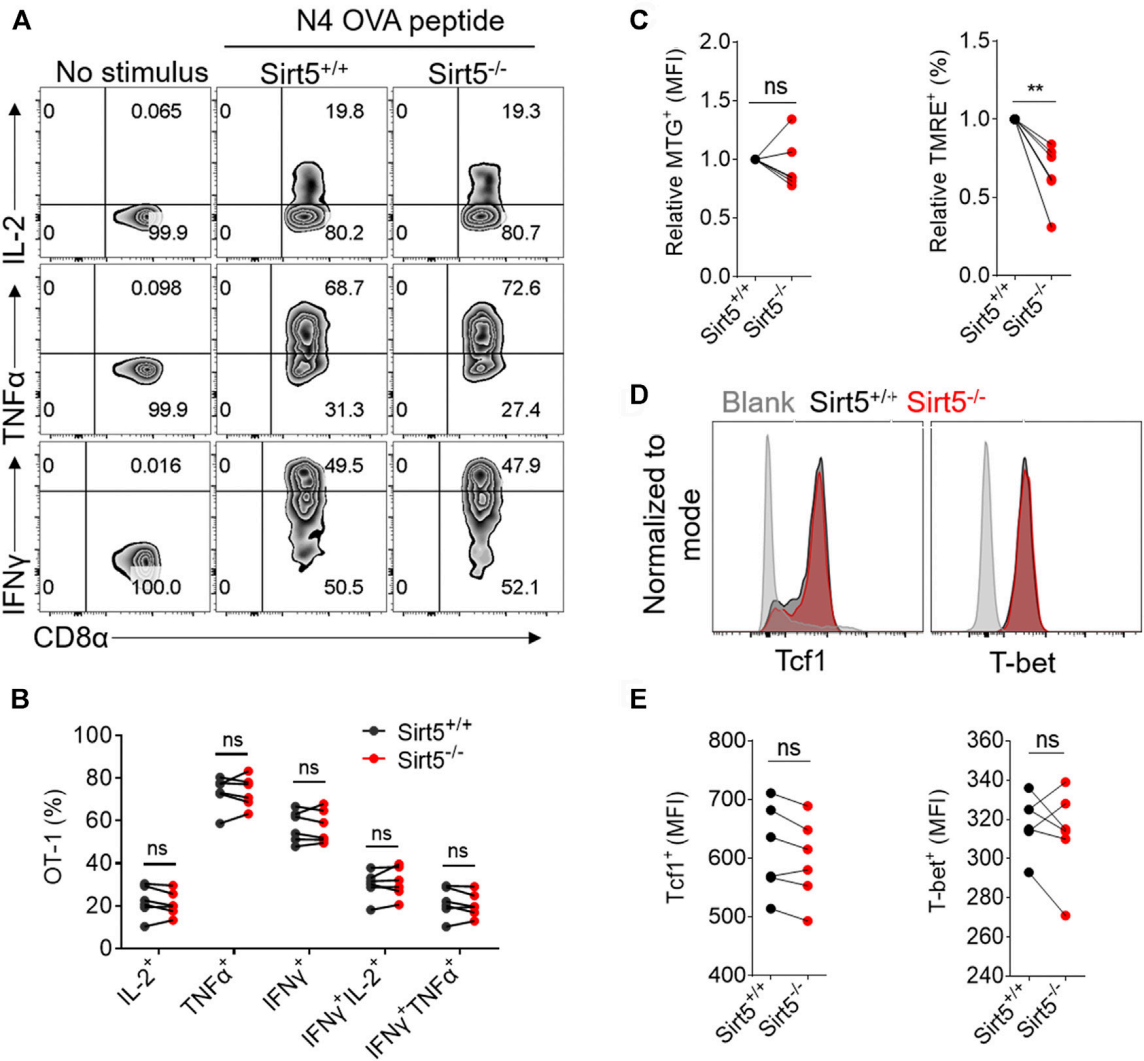
The effect of SIRT5 on the effector function, mitochondrial function and transcription factor expression of CD8^+^ T cells from the spleens of co-transferred mice. **(A)** Representative dot plots of IL-2, TNFα, and IFNγ expression levels of OT-1 cells from the spleen tissues by flow cytometry *in vitro* restimulation. The transferred mice are sacrificed on day 25 after secondary LM-OVA infection. **(B)** Statistical analysis of OT-1 cell ratio of cytokine experiment. Mean ± SD (*n* = 6), Student’s *t*-test. **(C)** Statistical analysis of relative MTG and TMRE MFI. Mean ± SD (*n* = 6), Student’s *t*-test, **: *p* < 0.01. **(D)** Representative histograms of Tcf1 and T-bet protein expression level of Sirt5^+/+^ and Sirt5^−/−^ OT-1 cells from the spleens. **(E)** Statistical analysis of Tcf1 and T-bet MFI. Mean ± SD (*n* = 6), Student’s *t*-test.

Memory CD8^+^ T cells exhibit quick expansion ability and strong effector function when encountering same antigen again, which is different from naïve CD8^+^ T cells. We thus measured the recall response of memory Sirt5^+/+^ and Sirt5^−/−^ OT-1 cells with the co-transfer model followed by secondary infection with relatively higher dose of bacteria (Figure 6A). The detection of blood samples demonstrated that SIRT5-deficient OT-1 cells did not show significant expansion advantage (Figures 6D,E). Consistently, Sirt5^−/−^ OT-1 cells did not show significant differences with that of Sirt5^+/+^ OT-1 cells in the survival in the spleens and lymphocyte nodes (Figures 6B,C). In addition, SIRT5 deficiency did not affect the differentiation of memory CD8^+^ T cells in the blood (seveth day), spleen, and lymphocyte nodes (25th day) (Figures 7A–F). Similar results were obtained in the blood on the 14th day and 21st day (data no shown). Moreover, regarding the cytokine production of IL-2, TNFα, and IFNγ and the protein expression of transcription factor Tcf1 and T-bet in the spleens and lymphocyte nodes, SIRT5 deficiency did not cause marked changes in the recall experiment (Figures 8A,B,D,E; Supplementary Figure S3). Of note, SIRT5 deficiency decreased the TMRE MFI of OT-1 cells (Figure 8C). Therefore, SIRT5 may be not required for the function, differentiation, and survival of memory CD8^+^ T cells upon secondary infection.

## Discussion

The roles of sirtuins in regulating CD8^+^ T cell differentiation and function are largely unknown, and it remains unclear whether SIRT5 regulates CD8^+^ T cell effector function and memory differentiation. Given that SIRT5 impacts on many mitochondrial enzyme activities and is involved in multiple cellular metabolism pathways, it is necessary to investigate the role of SIRT5 in CD8^+^ T cell effector or memory differentiation and functionality. Surprisingly, our results demonstrate that SIRT5 is dispensable for CD8^+^ T cell activation, proliferation, and transition to memory, and the survival and recall response of memory CD8^+^ T cells are also not dependent on SIRT5.

SIRT5 is currently the only enzyme known to possess demalonylase, desuccinylase, and deglutarylase activity and has been shown to exert multiple effects of desuccinaylation, demalonylation, and deglutarylation on intracellular biological pathways *via* modulating a large range of substrates, such as PKM2, isocitrate dehydrogenase 2 (IDH2), glucose-6-phosphate 1-dehydrogenase (G6PD), GAPDH, and carbamoyl-phosphate synthase (Tan et al., 2014; Nishida et al., 2015; Zhou et al., 2016; Wang et al., 2017). SIRT5 may play a role in regulating the development and function of immune cells by modulating their cellular mentalism pathways. Both Zhang et al. and Heinonen et al. have explored the innate immune responses of Sirt5^−/−^ mice to bacterial infections, respectively, but they obtained contradictory results due to utilization of the different mouse lines and bacterial strains (Heinonen et al., 2018; Zhang et al., 2020).

In this present study, although SIRT5 did not affect the activation process and cytokine secretion of CD8^+^ T cells *in vitro*, SIRT5 deficiency decreased the TMRE MFI (Figures 1E, 5C). The mitochondrial membrane potential is a crucial parameter affecting the T cell differentiation, as low-_△_ψm T cells exhibited memory phenotype with increased FAO and enhanced *in vivo* self-renewal and anti-tumor function (Sukumar et al., 2016). Thus, we transferred Sirt5^+/+^ and Sirt5^−/−^ OT-1 cells into the mice to monitor the *in vivo* dynamic changes. In the primary infection experiment, Sirt5^+/+^ and Sirt5^−/−^ OT-1 cells have comparable expansion, early memory differentiation capacity, and effector function in the blood, spleens, and lymphocyte nodes, regardless of separate-transfer or co-transfer. Next, we investigated the possibility that SIRT5 might affect the recall response, because memory T cells harbor specific metabolic or epigenetic programs to mount the recall response when encountered with same antigen. Yet, upon secondary infection, we observed similar expansion, survival capacity, differentiation, and effector function between Sirt5^+/+^ and Sirt5^−/−^ memory CD8^+^ T cells. Although Sirt5^−/−^ CD8^+^ T cells tended to have a slightly decreased mitochondrial membrane potential (Figure 8C), this SIRT5-induced TMRE decrement may be not sufficient for altering the immune response of CD8^+^ T cells. Tcf1 and T-bet are the two important transcription factors coordinately regulating the function and differentiation of CD8^+^ T cells; when Tcf1 is highly expressed in MPEC and Tcm cells, T-bet is highly upregulated in the SLEC cells (Pritchard et al., 2019; Zhao et al., 2021). However, comparable expression of Tcf1 and T-bet were observed between Sirt5^+/+^ and Sirt5^−/−^ OT-1 cells in our assay.

To this end, our observations that SIRT5 is not necessary for CD8^+^ T cell effector and memory differentiation are likely to be explained as follows. First, there exists a certain degree of functional overlap between SIRT5 and other sirtuin members. For example, SIRT3 protected calorie restriction on oxidative stress by deacetylating superoxide dismutase 2 (SOD2) and promoting its antioxidative activity (Qiu et al., 2010). SIRT3 altered the lipid metabolism of macrophages by targeting its deacetylated substrate IDH2 (Sheng et al., 2015). Similar to SIRT3, SIRT5 can deacylated SOD1, IHD1, and IDH2 and affected the oxidative stress and mitochondrial functions (Lin et al., 2013; Li et al., 2015; Zhou et al., 2016). As SIRT3 and SIRT5 share the similar subcellular location and targets, maybe due to their compensated functions, neither Sirt3 nor Sirt5 knockout altered host defenses against bacterial infection (Ciarlo et al., 2017; Heinonen et al., 2018), but dual deficiency of SIRT3 and SIRT5 exhibited a modest protection against listeriosis host defense (Heinonen et al., 2019). In addition, individual SIRT5 or SIRT3 deficiency did not lead to significant global metabolic abnormalities of mice (Fernandez-Marcos et al., 2012; Yu et al., 2013). Second, the functions of SIRT5 may be induced under specific conditions. Twenty-four of forty-eight hours of food withdraw significantly promoted the expression of SIRT5 in mouse hepatocytes (Ogura et al., 2010; Buler et al., 2014). Meanwhile, Yu et al. (2013) confirmed that, under steady state, SIRT5 was not necessary for the metabolic homeostasis. However, when exposed to extreme stress such as feeding with high-fat diet (HFD) for 12 months (not 1 week or 3 months), Sirt3^−/−^ mice displayed multiple metabolic syndromes including obesity, insulin resistance, hepatic steatosis, nonalcoholic steatohepatitis, or hyperlipidemias (Hirschey et al., 2011). During caloric restriction, SIRT3 modulated amino acid catabolism and β-oxidation by regulating ornithine transcarbamoylase activity and urea cycle (Hallows et al., 2011). Glucose limitation activated AMPK and its subsequent SENP1-SIRT3 signaling, promoting OXPHOS and mitochondrial fusion, which was beneficial to the anti-tumor immunity of T cells (He et al., 2021). Therefore, it remains unclear whether SIRT5 may play an important role under other physiological or pathological conditions, such as long time HFD, fasting, hypoxia, low glucose, low glutamine, or the tumor microenvironment. Third, CD8^+^ cells used in our experiments were derived from the whole-body Sirt5^−/−^ mice, in which the CD8^+^ cells may have adapted to SIRT5 deficiency throughout development. Immune cells including CD8^+^ T cells can reprogram their metabolism and adapt to the changes in the living environment to fulfill their biological functions (O'Neill and Pearce, 2016; Zhang and Romero, 2018). We cannot exclude this possibility that SIRT5 deletion triggers striking adaptation of CD8^+^ T cells. Therefore, the acute SIRT5 deletion with a conditional Cre recombinase or other SIRT5 deficiency/inhibition models such as retrovirus or lentivirus-mediated knockdown may provide valuable insights into CD8^+^ T cell functionality and differentiation due to the lack of systemic adaptations.

In summary, we reported here that CD8^+^ T cell memory differentiation and effector function were not impacted in the absence of SIRT5. Strikingly, the deletion of SIRT5 in CD8^+^ T cells did not lead to significant phenotypic changes through our *in vitro* and *in vivo* analysis, indicating that SIRT5 may be dispensable for differentiation and function of CD8^+^ T cells. Considering the redundant functions of other sirtuin family members that could compensate the deficiency of SIRT5 and the requirement of extreme induction condition in CD8^+^ T cells, we will focus our studies on the roles of SIRT5 in the CD8^+^ T cell immune response under specific conditions, such as the tumor microenvironment.

## Data Availability

The original contributions presented in the study are included in the article/Supplementary Material, further inquiries can be directed to the corresponding author.

## Supplementary Material

Supplementary Figure S1
**(A)** The mRNA expression of SIR5 in purified CD8^+^ T cell populations including naïve, effector, and memory CD8^+^ T cells, and PMs stimulated with LPS or not. The activated CD8^+^ T cells were further induced by IL-2/7 or IL-7/15 for 4 days, as indicated in Figure 1. In vivo memory CD8^+^ T cells were sorted from the spleens of OT-1 transferred mice on day 45 after LM-OVA infection. PMs were obtained as indicated in Figure 1 and were stimulated with LPS for 12 hours. **(B)** Surface activation marker expression of Sirt5^+/+^ and Sirt5^−/−^ OT-1 cells at the time points of 24 h and 6 days assessed by flow cytometry.

Supplementary Figure S2 The effect of SIRT5 on the cytokine secretion ability of CD8^+^ T cells from lymphocyte nodes of separate transferred mice. **(A)** Representative dot plots of IL-2, TNFα, and IFNγ expression levels of OT-1 cells from lymphocyte nodes by flow cytometry in vitro restimulation. The transferred mice are sacrificed on day 34 after primary LM-OVA infection. **(B)** Statistical analysis of OT-1 cell ratio of cytokine experiment. Mean ± SD (*n* = 9), Student’s t-test; ns, not significant.

Supplementary Figure S3 The effect of SIRT5 on the cytokine secretion ability of CD8^+^ T cells from lymphocyte nodes of co-transferred mice. **(A)** Representative dot plots of IL-2, TNFα, and IFNγ expression levels of OT-1 cells from lymphocyte nodes by flow cytometry in vitro restimulation. The transferred mice are sacrificed on day 25 after secondary LM-OVA infection. **(B)** Statistical analysis of OT-1 cell ratio of cytokine experiment. Mean ± SD (*n* = 6), Student’s t-test; ns, not significant.
