# Supplementary material for: Sirtuin 5 is Dispensable for CD8+ T Cell Effector and Memory Differentiation
Source: Front Cell Dev Biol. 2021 Dec 13;9:761193. doi: 10.3389/fcell.2021.761193 (PMC8710726; doi:10.3389/fcell.2021.761193)
Supplement: Supplementary file 1 [file Presentation1.pdf]

Sirtuin 5 is dispensable for CD8$^+$ T cell effector and memory differentiation

Qianqian Duan[1, 2, #], Jiying Ding[1, 2, 3, #], Fangfang Li[1, 2, 4], Xiaowei Liu[1, 2], Yunan Zhao[4], Hongxiu Yu[5], Yong Liu[6, 7, 8] and Lianjun Zhang[1, 2, 8, *]

[1]Institute of Systems Medicine, Chinese Academy of Medical Sciences & Peking Union Medical College, Beijing, China; [2]Suzhou Institute of Systems Medicine, Suzhou, Jiangsu 215123, China; [3]School of Life Science and Technology, China Pharmaceutical University, Nanjing, Jiangsu, China; [4]Institute of Biomedical Electromagnetic Engineering, Shenyang University of Technology, Shenyang, China; [5]Department of Systems Biology for Medicine, School of Basic Medical Sciences, Fudan University, Shanghai, China; [6]Cancer Institute, Xuzhou Medical University, Xuzhou, China; [7]Center of Clinical Oncology, The Affiliated Hospital of Xuzhou Medical University, Xuzhou, China; [8]Jiangsu Center for the Collaboration and Innovation of Cancer Biotherapy, Cancer Institute, Xuzhou Medical University, Xuzhou, China.

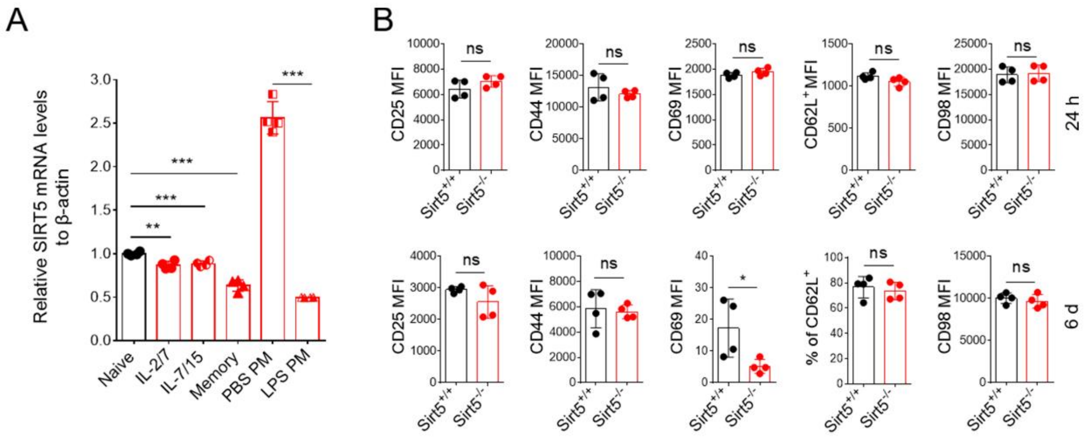

Supplementary Figure 1: (A) The mRNA expression of SIR5 in purified CD8+ T cell populations including naïve, effector, and memory $CD8^+$ T cells, and PMs stimulated with LPS or not. The activated $CD8^+$ T cells were further induced by IL-2/ 7 or IL-7/15 for 4 days, as indicated in Figure 1. In vivo memory $CD8^+$ T cells were sorted from the spleens of OT-1 transferred mice on day 45 after LM-OVA infection. PMs were obtained as indicated in Figure 1 and were stimulated with LPS for 12 hours. (B) Surface activation marker expression of $Sirt5^{+/+}$ and $Sirt5^{-/-}$ OT-1 cells at the time points of 24 h and 6 days assessed by flow cytometry.

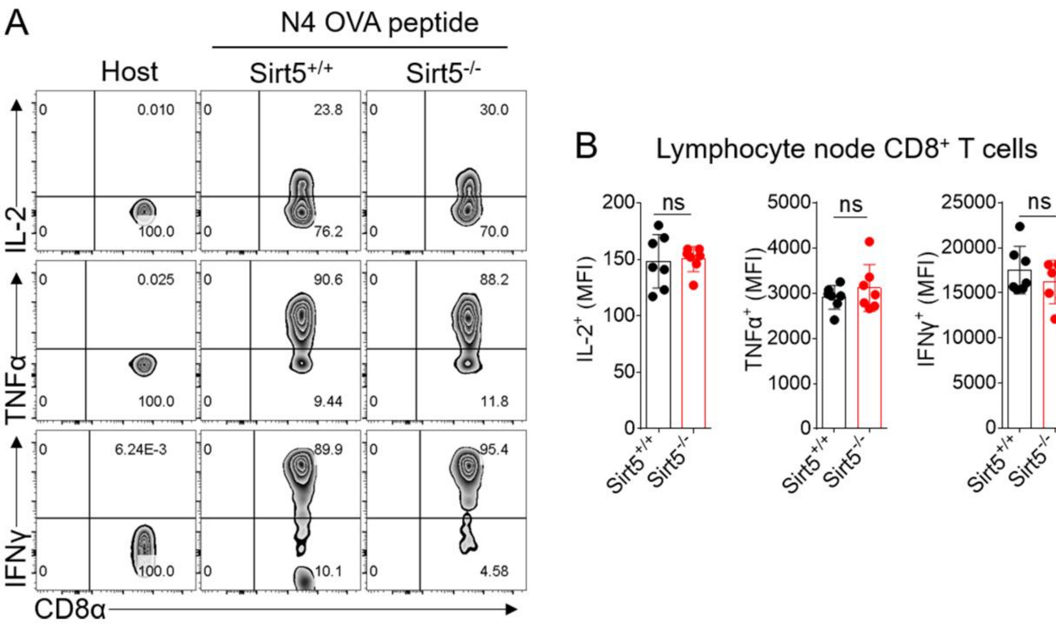

Supplementary Figure 2. The effect of SIRT5 on the cytokine secretion ability of $CD8^+$ T cells from lymphocyte nodes of separate transferred mice. (A) Representative dot plots of IL-2, TNFα, and IFNγ expression levels of OT-1 cells from lymphocyte nodes by flow cytometry in vitro restimulation. The transferred mice are sacrificed on day 34 post primary LM-OVA infection. (B) Statistical analysis of OT-1 cell ratio of cytokine experiment. Mean ± SD (n = 9), Student's *t*-test, ns: not significant.

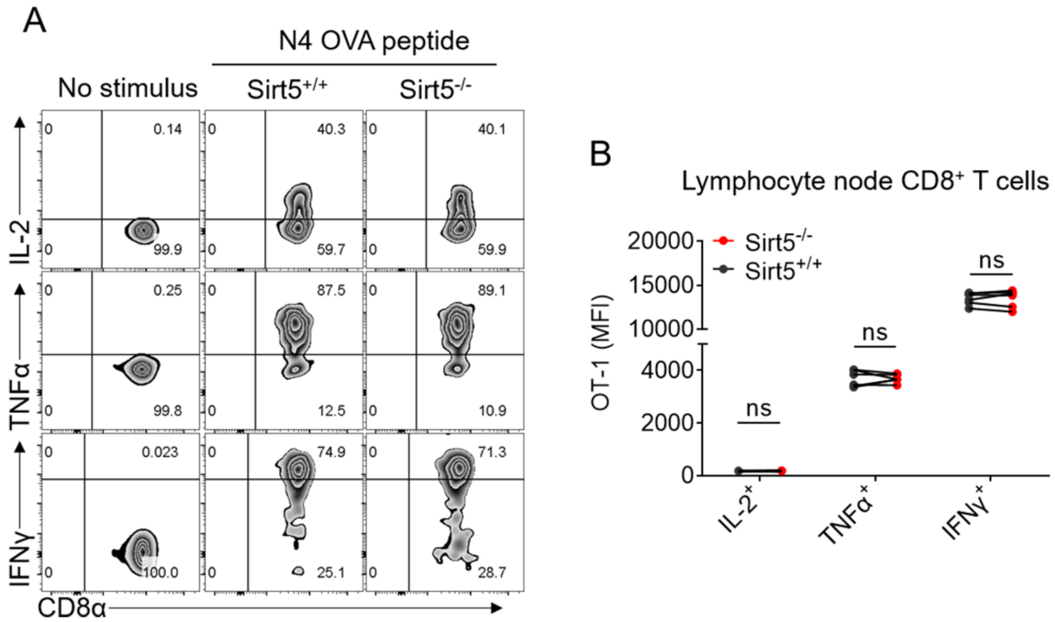

Supplementary Figure 3. The effect of SIRT5 on the cytokine secretion ability of CD8$^+$ T cells from lymphocyte nodes of co-transferred mice. (A) Representative dot plots of IL-2, TNFα, and IFNγ expression levels of OT-1 cells from lymphocyte nodes by flow cytometry in vitro restimulation. The transferred mice are sacrificed on day 25 post secondary LM-OVA infection. (B) Statistical analysis of OT-1 cell ratio of cytokine experiment. Mean ± SD (n = 6), Student's t-test, ns: not significant.
